# A Systemic Analysis of Transcriptomic and Epigenomic Data To Reveal Regulation Patterns for Complex Disease

**DOI:** 10.1534/g3.117.042408

**Published:** 2017-05-11

**Authors:** Chao Xu, Ji-Gang Zhang, Dongdong Lin, Lan Zhang, Hui Shen, Hong-Wen Deng

**Affiliations:** * Center of Genomics and Bioinformatics, Department of Global Biostatistics and Data Science, Tulane University, New Orleans, Louisiana 70112; † The Mind Research Network and Lovelace Biomedical and Environment Research Institute, Albuquerque, New Mexico 87106; ‡ Laboratory of Molecular and Statistical Genetics, Hunan Normal University, Changsha 410081, China

**Keywords:** integrative analysis, multi-omics data, sparse modeling, glioblastoma multiforme, network analysis

## Abstract

Integrating diverse genomics data can provide a global view of the complex biological processes related to the human complex diseases. Although substantial efforts have been made to integrate different omics data, there are at least three challenges for multi-omics integration methods: (i) How to simultaneously consider the effects of various genomic factors, since these factors jointly influence the phenotypes; (ii) How to effectively incorporate the information from publicly accessible databases and omics datasets to fully capture the interactions among (epi)genomic factors from diverse omics data; and (iii) Until present, the combination of more than two omics datasets has been poorly explored. Current integration approaches are not sufficient to address all of these challenges together. We proposed a novel integrative analysis framework by incorporating sparse model, multivariate analysis, Gaussian graphical model, and network analysis to address these three challenges simultaneously. Based on this strategy, we performed a systemic analysis for glioblastoma multiforme (GBM) integrating genome-wide gene expression, DNA methylation, and miRNA expression data. We identified three regulatory modules of genomic factors associated with GBM survival time and revealed a global regulatory pattern for GBM by combining the three modules, with respect to the common regulatory factors. Our method can not only identify disease-associated dysregulated genomic factors from different omics, but more importantly, it can incorporate the information from publicly accessible databases and omics datasets to infer a comprehensive interaction map of all these dysregulated genomic factors. Our work represents an innovative approach to enhance our understanding of molecular genomic mechanisms underlying human complex diseases.

Human complex diseases (*e.g.*, cancer) are induced by various genomic and epigenomic alterations in multiple biological processes (Wang *et al.* 2011). Studying a single type of biological data is generally insufficient to fully explore the underlying mechanisms of the human complex diseases. Recent advances in high-throughput technologies allow efficient investigation of various omics data, such as single nucleotide polymorphism (SNP), copy number variation (CNV), DNA methylation, and gene expression. Several pioneering studies have yielded genome-scale large datasets, including genomic, epigenomic, transcriptomic, and proteomic information, which are publicly accessible from large collaborative projects, including the ENCODE Project Consortium (2012), NIH Epigenomics Roadmap (Bernstein *et al.* 2010), and The Cancer Genome Atlas (TCGA) (Weinstein *et al.* 2013).

Concomitantly, the integration analyses of these diverse omics data are increasingly adopted to identify the potential causal (epi)genomic factors and ultimately provide the systematic view of fundamental insights into the complex mechanisms underlying the etiology of human diseases (Chen *et al.* 2014b; Hamed *et al.* 2015). For example, integrating genotype data with whole-genome gene expression data or DNA methylation data can identify the expression quantitative trait loci (eQTL) or the methylation quantitative trait loci (meQTL) (Shabalin 2012; Schadt *et al.* 2005). Methods for integrating gene expression data with miRNA, SNP, CNV, and DNA methylation data have been applied in cancer genomics (West *et al.* 2012; Taylor *et al.* 2009). Additionally, some latent variable models, such as canonical correlation analysis and partial least squares, were applied to identify the relationship between different omics datasets (Friedman *et al.* 2008; Zhao *et al.* 2012; Soneson *et al.* 2010). Recently, network analysis is increasingly gaining acceptance as a useful tool for data integration (Chen *et al.* 2014b; Kholodenko *et al.* 2012). Various network analysis methods have been developed to incorporate transcriptomic and proteomic data for computation of biological networks (Gosline *et al.* 2015; Wachter and Beissbarth 2015), to elucidate causality in biological networks (Gitter *et al.* 2011; Ourfali *et al.* 2007), and to integrate and visualize complex metabolomics results even in cases where biochemical domain knowledge or molecular annotations are unknown (Grapov *et al.* 2015; Karnovsky *et al.* 2012).

Although substantial efforts have been made to integrate different omics data, there are at least three challenges for multi-omics integration methods to overcome: (i) How to simultaneously consider the effects of all kinds of genomic and epigenomic factors, since these factors jointly influence the phenotypes (Lander 2011); (ii) How to effectively incorporate the information from publicly accessible databases and omics datasets to fully capture the interactions among (epi)genomic factors from diverse omics data sources; and (iii) Until present, the combination of more than two omics datasets has been poorly explored compared with those that intend to integrate two various omics datasets, such as those in eQTL and meQTL analyses (Pineda *et al.* 2015). Current integration approaches are not sufficient to address all of these challenges simultaneously in one analytical framework.

To address these three challenges in multi-omics integration analysis, we presented a novel integrative analysis framework that incorporated sparse model, multivariate analysis, Gaussian graphical model (GGM), and network analysis. Our method can not only simultaneously identify disease-associated (epi)genomic factors from diverse omics data, but also incorporate the information from publicly accessible databases and omics datasets to infer the regulatory modules of these (epi)genomic factors. By applying this strategy to systemically study the genome-wide gene expression, DNA methylation, and miRNA expression data of glioblastoma multiforme (GBM) samples, we identified three regulatory modules of dysregulated (epi)genomic factors associated with GBM patient survival time. By combining the three regulatory modules with respect to the common regulatory factors, we further revealed a promising global regulatory pattern critical for GBM survival. Our integrative analysis represents an innovative approach to enhance our comprehensive understanding of molecular genomic mechanisms underlying human complex diseases.

## Methods

It is well known that genes usually jointly contribute to certain diseases and many epigenomic factors play an important role in the development of complex diseases by regulating gene expression. Our goal is to infer a comprehensive interaction map of all these dysregulated (epi)genomic factors.

**Figure 1:**
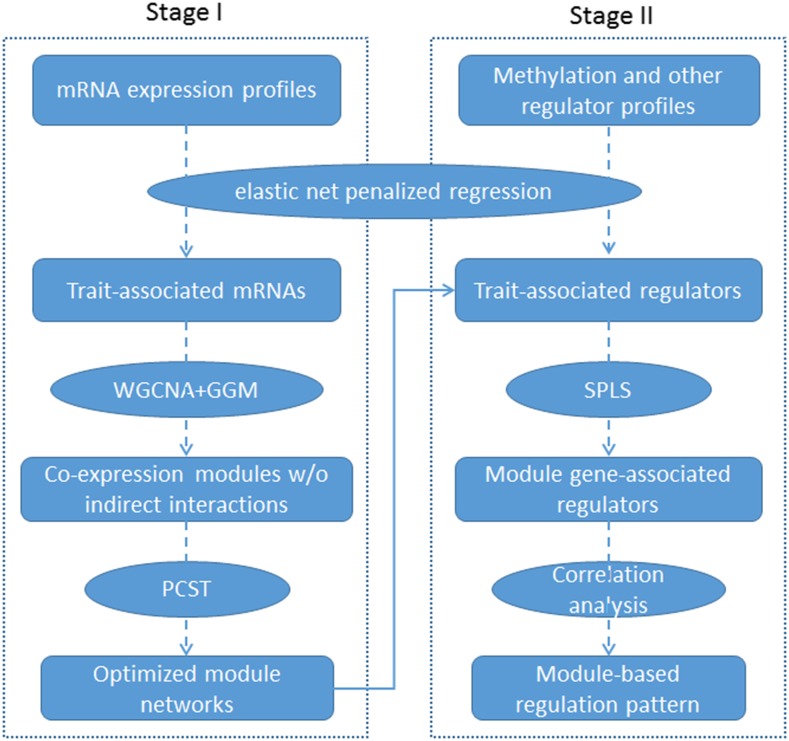
Workflow of the integrative analysis of multi-omics data.

Thus, we divided our integrative analysis into two major stages as shown in Figure 1: we first identify the trait-related mRNAs and build optimized coexpression modules with these mRNAs, and then infer regulation pattern between the epigenomic (and/or other omics factors) and the identified coexpression module.

### Stage 1: build optimized mRNA coexpression module networks associated with the trait of interest

Considering the different gene functional modules may contribute to certain diseases and the computational burden for the downstream analysis, we propose to identify trait-associated mRNAs and subsequently discover the coexpression modules with trait-associated mRNAs through coexpression network analysis.

First, we apply the elastic net penalized regression model to select a set of trait-associated mRNAs. The elastic net is particularly useful to handle the situation of small sample size and a large number of features. In addition, it encourages the selection of strongly correlated predictors in or out of the model together, which is helpful to preserve the information for the following module identification (Zou and Hastie 2005). The elastic net penalized regression model is illustrated as follows:

$$\hat{\beta }=\arg\;\underset{\beta }{\min}L\left(\beta \right)+\sum_{k=1}^{p}\lambda \left[\alpha \left|\beta_{k}\right|+\left(1-\alpha \right)\beta_{k}^{2}\right],$$

where the regularization parameter *λ* > 0 controls the overall strength of the penalty and 0 < *α* ≤ 1 bridges the gap between lasso (*α* = 1, the default) and ridge (*α* = 0) penalty. L(β) is the loss function given a fitted model, such as the residual sum of squares for the ordinary linear model or the negative log partial likelihood function for the Cox’s proportional hazards model. The optimal *λ* and *α* can be chosen by 10-fold cross-validation.

The trait-associated mRNAs are then subjected to weighted correlation network analysis (WGCNA) (Zhang and Horvath 2005) for the identification of high coexpression modules, denoted as $M=\left\{M_{i},i=1,2,..,n_{m}\right\},$ where $n_{m}$ is the number of modules identified. Computations are carried out using the R package WGCNA (Zhang and Horvath 2005). The relationships stored in the coexpression modules include direct interactions, which connects one pair of genes directly, and indirect interactions, where two genes are connected due to a path with multiple edges (Poyatos 2011). We then remove the indirect interactions in the coexpression modules through a partial correlation analysis.

The GGM reveals direct associations with conditional independence/dependence among variables using partial correlation coeffcients (Mader *et al.* 2015). Given a coexpression module $M_{i},$ it is assumed the expression of the genes in $M_{i}$ follows a multivariate Gaussian distribution with mean *μ* and covariance matrix Σ. The conditional independence between *g_i_* and *g_j_* given the other genes *g*_− {_*_ij_*_}_, denoted by P(*g_i_*, *g_j_*|*g*_−{_*_ij_*_}_) = P(*g_i_*|*g*_− {_*_ij_*_}_) P(*g_j_*|*g*_− {_*_ij_*_}_), is equivalent to that the corresponding element in the precision matrix is zero (Wang and Huang 2014), *i.e.*, *ω_ij_* = 0. The precision matrix is the inverse of the covariance matrix of genes in $M_{i},$ denoted by Ω = (*ω_ij_*) = Σ^−1^. A partial correlation P(*g_i_*, *g_j_*|*g*_− {_*_ij_*_}_) is formally written as ${\hat{\gamma }}_{ij}=-{\hat{\omega }}_{ij}/\sqrt{{\hat{\omega }}_{ii}{\hat{\omega }}_{jj}}$ with the property $\sqrt{n{\left(1-{\hat{\gamma }}_{ij}^{2}\right)}^{-2}}\left({\hat{\gamma }}_{ij}-\gamma_{ij}\right)\overset{D}{\to }N\left(0,1\right),$ where ${\hat{\omega }}_{ij},$ ${\hat{\omega }}_{ii}$, and ${\hat{\omega }}_{jj}$ are the estimators of $\omega_{ii},$ $\omega_{ij}$, and $\omega_{jj}$ (Wang *et al.* 2016). Only the edges with ${\hat{\gamma }}_{ij}$ significantly different from zero will be preserved. An FDR of 0.05 is used as the cut-off for statistical significance to adjust for the multiple testing. The filtered modules are denoted by $M^{\prime }=\left\{M_{i}^{\prime },i=1,2,..,n_{m}\right\}.$

After removing the indirect interactions, an optimal subnetwork is refined that which may play an important role on the trait of interest. Given one module is $M_{i}^{\prime }=\left(V,E\right)$, with a node set as V and direct interactions set as E, the Prize Collecting Steiner Tree (PCST) algorithm, which is able to reconstruct compact networks of the functionally relevant connections with control of the false positives in the network (Huang and Fraenkel 2009), is used to find a set of most confident interactions that connect the terminal genes in the network $M_{i}^{\prime \prime }=\left(V^{\prime },E^{\prime }\right)$, using the following function that simultaneously minimizes the cost of edges included and the penalties of nodes excluded:

$$M_{i}^{\prime \prime }=\underset{\begin{array}{c} E^{\prime }\subseteq E;\;V^{\prime }\subseteq V\\ \left(E^{\prime },\;V^{\prime }\right)\;\mathrm{connected} \end{array}}{\min}\left(\sum_{e\in E^{\prime }}c_{e}-\sum_{i\in V^{\prime }}b_{i}\right)$$

$$\text{with}c_{e}=1-\prod_{j}^{k}r_{j},$$

where the node prize *b_i_* is the weight of node *i*; *c_e_* is the cost of edges with *k* interaction evidence, *r_j_* indicates the score [0, 1] of the *j*th interaction evidence. The weight of node *i* is from the univariate mRNA trait regression analysis. We assign the absolute value of the regression coefficient of the node *i* to *b_i_*. The cost of edges is derived from two kinds of evidence: (i) information from publicly accessible databases and omics datasets, such as the Search Tool for the Retrieval of Interacting Genes (STRING) database (http://string-db.org/), or public datasets; and (ii) correlations between nodes in the local datasets. Instead of using the standard definition of $c_{e},$ we combine the two kinds of evidence into the edge weight using $c_{e}=1-S,$ where

$$S=\left[1-\prod_{j}\left(1-\frac{S_{j}-p}{1-p}\right)\right]\left(1-p\right)+p,$$

a naive Bayesian approach to measure the interaction evidence among nodes (Szklarczyk *et al.* 2011). $S_{j}\in \left[0,1\right]$ is the subscore downloaded from STRING or the absolute value of the correlations from the local datasets. This method integrates the scores by multiplying the probabilities of associations not predicting a functional interaction while adjusting for the prior probability (p) for any two genes being linked, which is 0.063 according to the KEGG benchmark dataset (Franceschini *et al.* 2013). In the calculation, the prior corrected score is constrained to be within [0, 1] (see the source code online). Compared with the standard method, it yields higher confidence when more than one type of evidence supports a given interaction (von Mering *et al.* 2003).

### Stage 2: infer module-based regulation pattern with epigenomic and/or other omics data

To identify DNA methylation sites and other factors that potentially regulate the optimized module subnetwork $M_{i}^{\prime \prime }$ (such as miRNAs), we examine their associations with the genes within $M_{i}^{\prime \prime }.$ Let $Y\in R^{n\times p}$ be the data matrix of $M_{i}^{\prime \prime }$ derived in stage 1, where *n* is the number of patients with complete phenotypic and multi-omics data, and *p* is the number of mRNAs in the given module $M_{i}^{\prime \prime }.$

First, we screened trait-associated methylation sites and other regulators by the elastic net penalized regression model as in the analyses of mRNAs in stage 1. Let $D\in R^{n\times q}$ be the combined data matrix for the *q* trait-associated regulators, which may include methylation sites and other factors. To deal with the large number of features and small sample size, we use the sparse partial least square (SPLS) regression model to identify the regulators that are correlated with the module $M_{i}^{\prime \prime }$ (Meng *et al.* 2016):

$$\max\;\mathrm{cov}\left(Y\alpha ,D\;\beta \right)+\lambda_{1}\left[\Phi \left(\alpha \right)\right]+\lambda_{2}\left[\Phi \left(\beta \right)\right]$$

$$s.t.\;\beta^{T}\beta =1,\alpha^{T}\alpha =1,$$

where α, *β* are loading vectors for the latent vectors E=Yα,Γ=Dβ, respectively. The sparse regularization function Φ(⋅) including L1 and L2 penalties is imposed on α, *β*: the L1 penalty is applied to set the coefficients of the irrelevant variables to 0; the L2 penalty is added to handle multicollinearity among covariates (Chun and Keles 2010; Chun *et al.* 2011). The optimal regularization parameter can be chosen by 10-fold cross-validation (Chun *et al.* 2011). The resulted regulators associated with the module $M_{i}^{\prime \prime }$ are denoted by $R_{i}.$

To reveal the module-based regulation patterns, the Pearson’s correlation is used to detect correlations between the identified regulators $R_{i}$ and mRNAs within the module $M_{i}^{\prime \prime }.$ The cut-off of correlation test *p* value ≤0.05 (*t*-test) is applied to select those regulator mRNA pairs of potential interest. Those chosen pairs are further filtered by external databases if there are any, *i.e.*, Exiqon miRSearch, TargetScan, and microRNA.org for miRNA–mRNA regulatory relationships (Chen and Rajewsky 2007; Akhtar *et al.* 2016).

### Data availability

The source code in this study is publicly available at https://github.com/xu1912/SMON.git.

## Results

**Figure 2:**
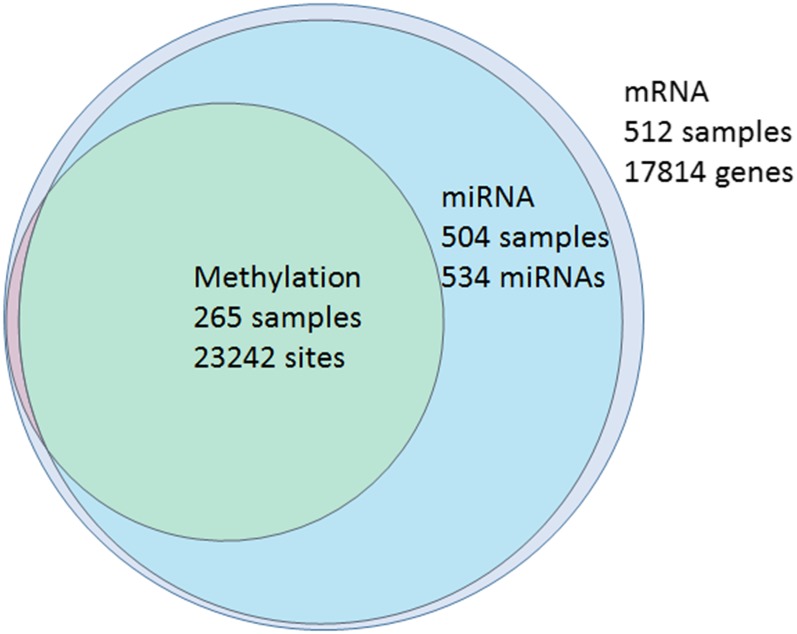
Venn diagram of the samples with different Omics data. Methylation ∩ miRNA = 261; methylation ∩ mRNA = 265; miRNA ∩ mRNA = 504.

Here, we apply our framework to integrate three different omics datasets (mRNA expression, miRNA expression, and DNA methylation) from the GBM study. All normalized data (level 3) were collected from The Cancer Genome Atlas (TCGA) portal, and can be accessed from the TCGA-GBM project repository at National Cancer Institute Genomic Data Commons Data Portal (https://portal.gdc.cancer.gov/legacy-archive/search/f). To minimize the scale differences among different omics data, the features of the three omics datasets were standardized to have zero means and unit SDs. The clinical outcome of interest is the patient survival time. From the GBM datasets, we excluded those patients whose survival time was <30 d to remove short survival due to reasons such as postoperative complications from the surgery (Kim *et al.* 2010). Figure 2 presents the sample size of the three omics datasets and the overlapping samples among them.

Identifying trait-associated genes in mRNA data are the first step in reconstructing trait-associated coexpression modules. As detailed in the *Methods* section, the sparse Cox’s proportional hazards model was used to select genes associated with patients’ survival time, which can simultaneously incorporate thousands of genomic markers working collaboratively with joint effects on the trait of interest in a single statistical model. After 10-fold cross-validation, we identified 217 genes with 512 subjects in mRNA data significantly associated with GBM survival time, including numerous GBM-related genes such as *FZD7* (Kierulf-Vieira *et al.* 2016), *TPPP3* (Fomchenko *et al.* 2011), and *LGALS3* (Ma *et al.* 2014). Using the Dynamic Tree Cut method in WGCNA, we identified three coexpression modules, which are composed of 50, 28, and 18 genes, respectively. There were 121 genes (55.76% in total identified genes) that were not grouped in any community. This was partially due to the minimum size of modules we set. In this study, the minimum size of modules was set as 15, which means those genes were not assigned in a module due to their size being <15, even if they were grouped.

**Figure 3:**
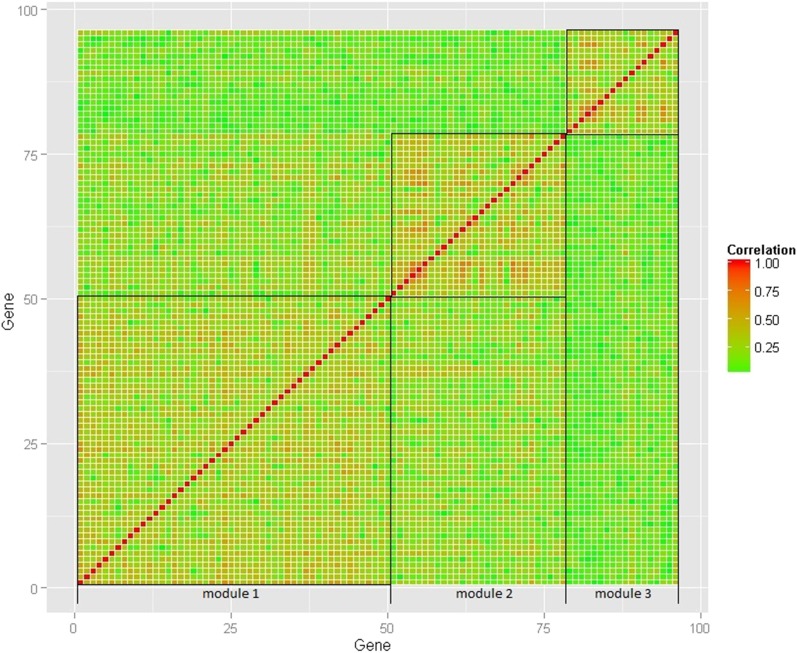
Heat map of correlations between and within coexpression modules constructed by WGCNA. Each row/column represents a gene. Each cell element is the absolute value of correlation coefficient between two genes. The intensity of red coloring indicates the strength of correlation between pairs of genes, with green color corresponding to low correlation. The independent modules are represented as isolated boxes along the diagonal.

Figure 3 presents the correlation heat map of modules constructed through WGCNA. In the heat map, each row (column) corresponds to a gene. The independent modules are represented as isolated boxes along the diagonal. Inspecting the correlation between and within the module memberships, these genes within each module are found to be strongly connected (reflected by the majority of red blocks within each module in Figure 3), and the genes between modules show weak connections (reflected by the overall green block in the heat map in Figure 3). The weak interconnectivity between modules suggests that the three modules may function separately and affect patient survival time in relatively independent ways.

We estimated the partial correlations between genes in each module to trim indirect interactions. In each module, we selected significant gene pairs (FDR ≤ 0.05) for their partial correlations and reconstructed the coexpression networks using these significant gene pairs. As shown in Supplemental Material, Figure S1, in modules 1–3, we identified 272, 141, and 141 gene pairs with significant partial correlations, respectively, and trimmed 77.80, 62.70, and 7.84% of gene pairs as indirect interactions. In addition, by incorporating the information on protein–protein interactions (PPIs) from the STRING database, we further filtered the unreliable or indirect interactions in the modules by PCST. In total, 49 gene pairs from module 1, 27 pairs from module 2, and 17 pairs from module 3 were determined as the most reliable interactions (Figure S1) that had multiple lines of evidence supporting their potential functionality in the cell. We also performed pathway enrichment analysis on the genes collected from all the three modules with WEB-based GEne SeT AnaLysis Toolkit (Wang *et al.* 2013) to investigate how well the modules functioned in a GBM-related process, as annotated by KEGG database (Kanehisa *et al.* 2012). An additional file (Table S1) lists the nine most significant KEGG pathways with adjusted *p* values <0.05, including mRNA surveillance pathway, pathways in cancer, *WNT* signaling pathway, *etc*. Among these pathways, it is well known that *WNT* signaling pathway regulates proliferation, death, and migration and cell fate decision. Dysregulation of the *WNT* signaling pathway was associated with various cancers, including GBM (Lee *et al.* 2016). The members *FZD7* and *FZD10* in the *WNT* signaling pathway are important receptors. In many types of cancer, *FZD2* expression was strongly correlated with poor prognosis (Mine *et al.* 2015). Therefore, our results may reveal the regulation pattern of *FZD2* and *FZD10* expression by network analysis, which could be utilized for epigenomic-based therapy for GBM.

To incorporate DNA methylation and miRNA data into the coexpression modules, we identified miRNAs and methylation sites that are associated with patient survival time, and then used SPLS regression to determine those miRNAs and DNA methylation sites that are also associated with the genes of the three coexpression modules in Table 1. Further, within each module, we determined genes with *cis*-correlated DNA methylation sites, as well as miRNA–mRNA pairs.

**Table 1:** The identified miRNAs and methylation sites for three modules by SPLS model

	mRNAs	miRNAs	Methylation Sites
Module 1	50	46	353
Module 2	28	11	125
Module 3	18	33	174

**Figure 4:**
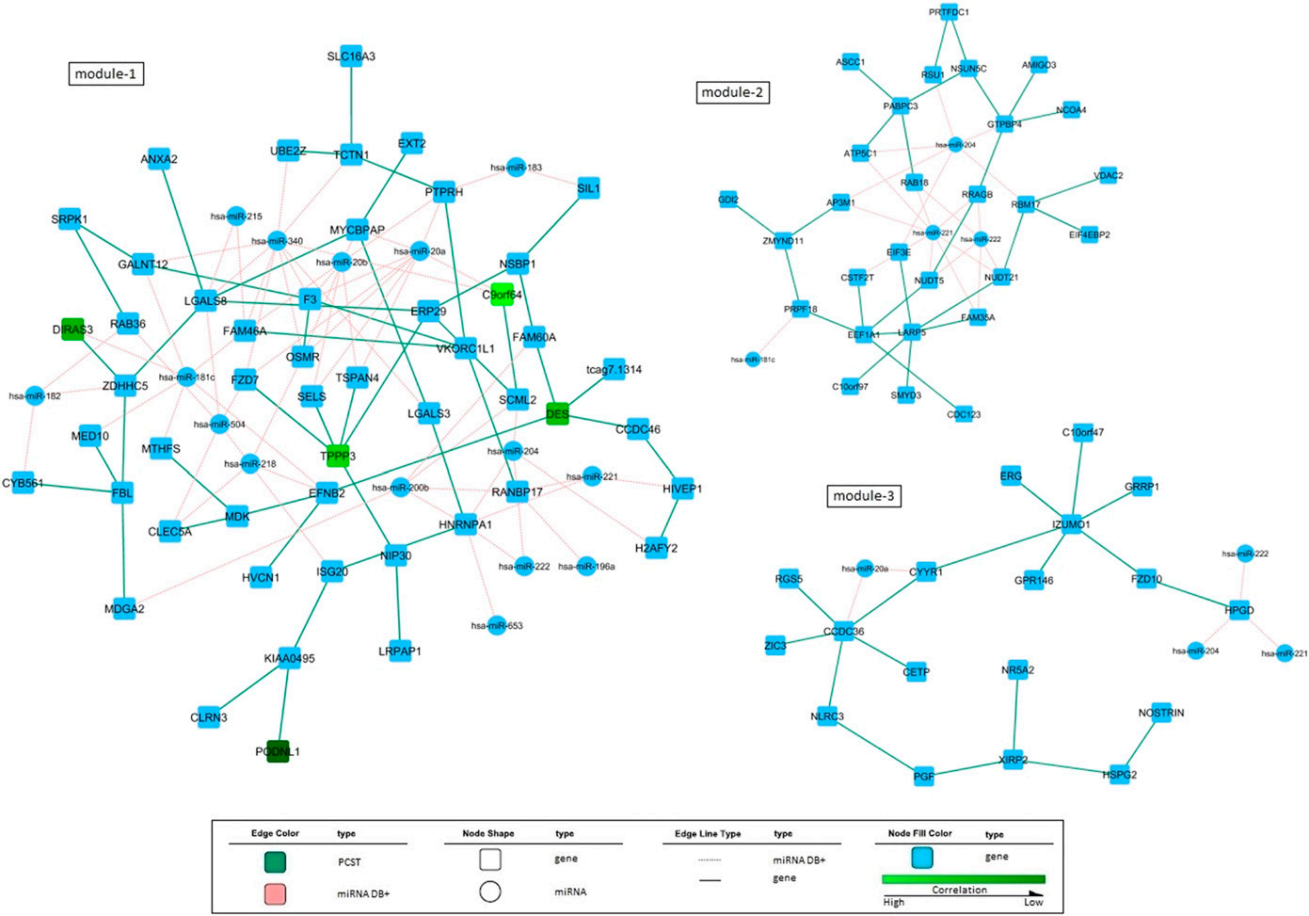
The interaction modules incorporating information of miRNAs and methylation sites. Each rectangle represents a gene; the circle represents miRNA. The green rectangles are genes with *cis* effects, with brighter indicating higher. The pink dashed edges indicate miRNA–gene interactions annotated in previously mentioned miRNA databases. The green solid edges are gene–gene connections resulting from PCST.

It is known that DNA methylation is an important epigenomic mechanism to regulate gene expression. If there is a significant association for one gene between its methylation level and expression level, it is called *cis* relationship; otherwise, it is defined as *trans* relationship (Smith and Meissner 2013). Since the reproducibility of *trans* relationships is still in debate, we focused on genes with *cis* relationships (van Nas *et al.* 2010; van Eijk *et al.* 2012). It can be seen that only four gene sites in module 1 have *cis* effects (Figure 4). The methylation levels of these four gene sites show negative correlations with the expression levels of corresponding genes. In modules 2 and 3, we did not identify the gene sites with *cis* effects on corresponding genes.

miRNA is well known for the major function of cleaving transcripts of its target genes at the post-transcriptional level (He and Hannon 2004). Thus, we were most interested in a negative correlation between miRNA and gene expression. The external databases Exiqon miRSearch, TargetScan, and microRNA.org were used to filter the miRNA–mRNA pairs with miRNA–target regulatory relationships (Chen and Rajewsky 2007; Akhtar *et al.* 2016). Those miRNA–mRNA interactions with significant negative correlations and miRNA–target relationships are kept. As shown in Figure 4, we identified 15 miRNAs for module 1, four for module 2, and four for module 3.

Our results highlight a number of interesting regulatory mechanisms that may be critical for GBM development and progress. For example, our results suggest that *miRNA-181c* and the methylation level of *DIRAS3* both contribute to the alteration of *DIRAS3* expression (module 1 in Figure 4), which may, in turn, affect the GBM survival time. *DIRAS3* (also known as *ARHI*) is a known tumor suppressor gene and overexpression of *DIRAS3* resulted in suppression of glioma cell proliferation, arrest of cell-cycle progression, reduction in cell migration and invasion, and promotion of cell apoptosis (Chen *et al.* 2014a). In addition, *miRNA-181c* was reported as a tumor-related gene in glioma cells (Ruan *et al.* 2015). Thus, our results indicate one possible regulation mechanism of these tumor-related factors and may provide candidate targets for gene therapy of glioma.

**Figure 5:**
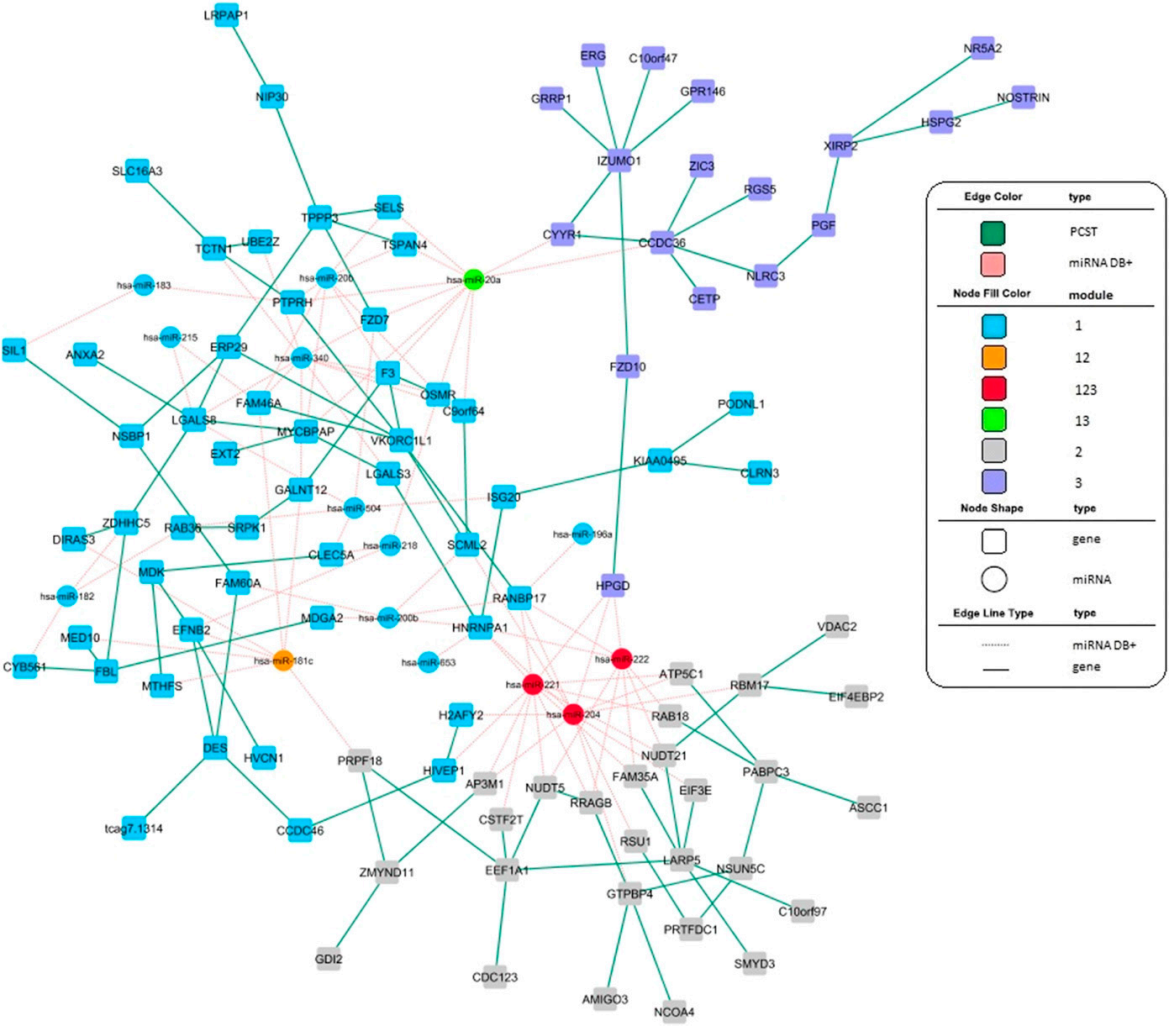
Combined regulatory network with three identified modules. Each rectangle represents a gene; the circle represents miRNA. Different colors of the rectangles/circles indicate their belonging to a single module or overlap of the three modules. The pink dashed edges indicate miRNA–gene interactions annotated in previously mentioned miRNA databases. The green solid edges are gene–gene connections resulting from PCST.

We combined the three modules to have a global view (Figure 5) of the regulatory networks contributing to GBM patient survival time. The top six miRNAs with most edges in the combined network were *miRNA-221*, *miRNA-204*, *miRNA-20a*, *miRNA-340*, *miRNA-222*, and *miRNA-181c*. Among these six miRNAs, *miRNA-221*, *-204*, and *-222* were shared by three modules; *miRNA-181c* was shared by modules 1 and 2; *miRNA-20a* was shared by modules 1 and 3; and *miRNA-340* was not shared among modules. These miRNAs may mediate cooperative regulation of different modules and thus may play particularly critical roles in regulating GBM development and progress. *miRNA-221* and *miRNA-222* are oncogenic miRNAs that have been studied in relation to a diverse list of cancers, including GBM. When overexpressed *in vitro*, both *miRNA-221* and *miRNA-222* potentiate classic cancer hallmarks, *i.e.*, proliferation, angiogenesis, and invasion (Henriksen *et al.* 2014; Singh *et al.* 2012; Zhang *et al.* 2009). Due to their broader functional relevance, *miRNA-20a*, *-204*, *-181c*, and *-340* also have been identified as oncogenic genes and may serve as targets for treatment of GBM (Wang *et al.* 2015; Wei *et al.* 2015; Xia *et al.* 2015; Huang *et al.* 2015; Ruan *et al.* 2015). Additionally, several target genes of these miRNAs have been validated in previous studies, *e.g.*, *RAB18*, *RSU1*, *GTPBP4*, *DIRAS3*, and *F3* (Behrends *et al.* 2003; Chunduru *et al.* 2002; Lee *et al.* 2007; Riemenschneider *et al.* 2008; Gessler *et al.* 2010). Taken together, our findings highlight several miRNAs that may regulate multiple signaling cascades crucial for gliomagenesis and therefore, these miRNAs could be therapeutically significant.

## Discussion

Single-omics studies (genome/transcriptome/epigenome/proteome) fall short of illuminating the underlying functional mechanisms and providing a comprehensive view of the regulatory patterns of genomic factors across multiple omics datasets for the etiology of human diseases (Farber and Lusis 2009). Integrating multi-omics datasets in network analysis may facilitate the discovery of novel susceptibility genes for human complex diseases, and more importantly, yield a comprehensive understanding of the complex regulatory mechanisms embedded in and across multi-omics data (Farber 2010). In this study, we proposed an integrative network analysis framework with epigenomic and transcriptomic data to identify regulatory patterns relevant to the trait of interest. The additional analysis in File S1 indicates that our framework can produce reliable results.

Our framework started with the coexpression network analysis to identify the coexpression modules based on the following two considerations: (i) due to the complexity of human diseases, it is highly likely that different gene functional modules may contribute to certain diseases, and since different modules tend to have different biological functions, it is reasonable to analyze each module separately in the sense that different biological functionalities should be considered separately (Ma *et al.* 2012); (ii) a benefit of coexpression network analysis is that it can greatly decrease the computational burden for the downstream analysis, *e.g.*, partial correlation analysis and inference of optimal subnetworks. For example, if we have 300 trait-associated genes, there will be 44,850 gene pairs to be tested in partial correlation analysis, and this number will increase dramatically with the increase of the number of trait-associated genes, which will lead to heavy computation burden and decrease of power to identify gene pairs with significant partial correlations.

In the case of complex diseases (such as GBM), comprehensively identifying interactions among (epi)genomic factors is important to systematically dissect cellular roles of those (epi)genomic factors and to gain insights into metabolic pathways. With a coexpression network, the number of correlations is generally considerably high, suggesting a plethora of indirect interactions (Krumsiek *et al.* 2011). To remove indirect interactions among genomic factors and refer reliable regulation networks, our proposed method incorporated two kinds of analyses for a given coexpression module: partial correlation analysis and inference of optimal subnetworks. In partial correlation analysis, GGMs were applied to distinguish direct from indirect associations by estimating the conditional dependence between genes based on partial correlation coefficients. However, conditional independence by itself is insufficient to remove all indirect relationships. Thus, compiling the information from external public databases will be helpful to further prune those unlikely, indirect, and spurious interactions. We can retrieve various genomic interactions from many available public databases and multi-omics datasets, such as PPIs. In this study, we incorporated interaction information from PPIs and the results of the partial correlation analysis to compute a score as a confidence score for each interaction in the module. The most reliable interactions of each module were further inferred through searching optimal subnetwork for the given module.

Since our goal is to identify regulatory patterns relevant to the trait of interest from epigenomic and transcriptomic data, it is reasonable to only choose the trait-associated (epi)genomic factors from multi-omics data for the subsequent network analysis. This selection can not only remove noise but also decrease the computational cost in the network construction of multi-omics data. During the selection procedure, we adopted a sparse model using L1 and L2 penalties to identify the trait-associated genomic factors, which has the following specific advantages: (i) it accommodates tens of thousands of features at a time and identifies joint effects of a combination of trait-associated genomic factors, including those with small effect sizes; (ii) using both L1 and L2 penalties, it is able to select groups of correlated variables, which are very common in high-dimensional genomic data. The selection of relevant correlated genomic factors is essentially important for coexpression network analysis and discovery of regulation patterns. Thus, the sparse model using both L1 and L2 penalties demonstrates the efficiency in feature selection and captures informative genomic factors.

In summary, our method can not only identify disease-associated dysregulated genomic factors, but also, more importantly, construct a comprehensive map of interactions of all these dysregulated genomic factors implicated in a specific disease. It is essential to understand the intricacy of the genomic mechanisms behind complex diseases, and this may support the development of new therapeutics. However, it should be recognized that network representation of the complexity of biological systems is just the beginning. This study is expected to pioneer an innovative approach to comprehensively enhance our understanding of molecular genomic mechanism in human complex diseases.

## Supplementary Material

Supplemental material is available online at www.g3journal.org/lookup/suppl/doi:10.1534/g3.117.042408/-/DC1.
