# Supplementary material for: A Systemic Analysis of Transcriptomic and Epigenomic Data To Reveal Regulation Patterns for Complex Disease
Source: G3 (Bethesda). 2017 May 11;7(7):2271–9. doi: 10.1534/g3.117.042408 (PMC5499134; doi:10.1534/g3.117.042408)
Supplement: Supplementary file 1 [file 2271FigureS1.docx]

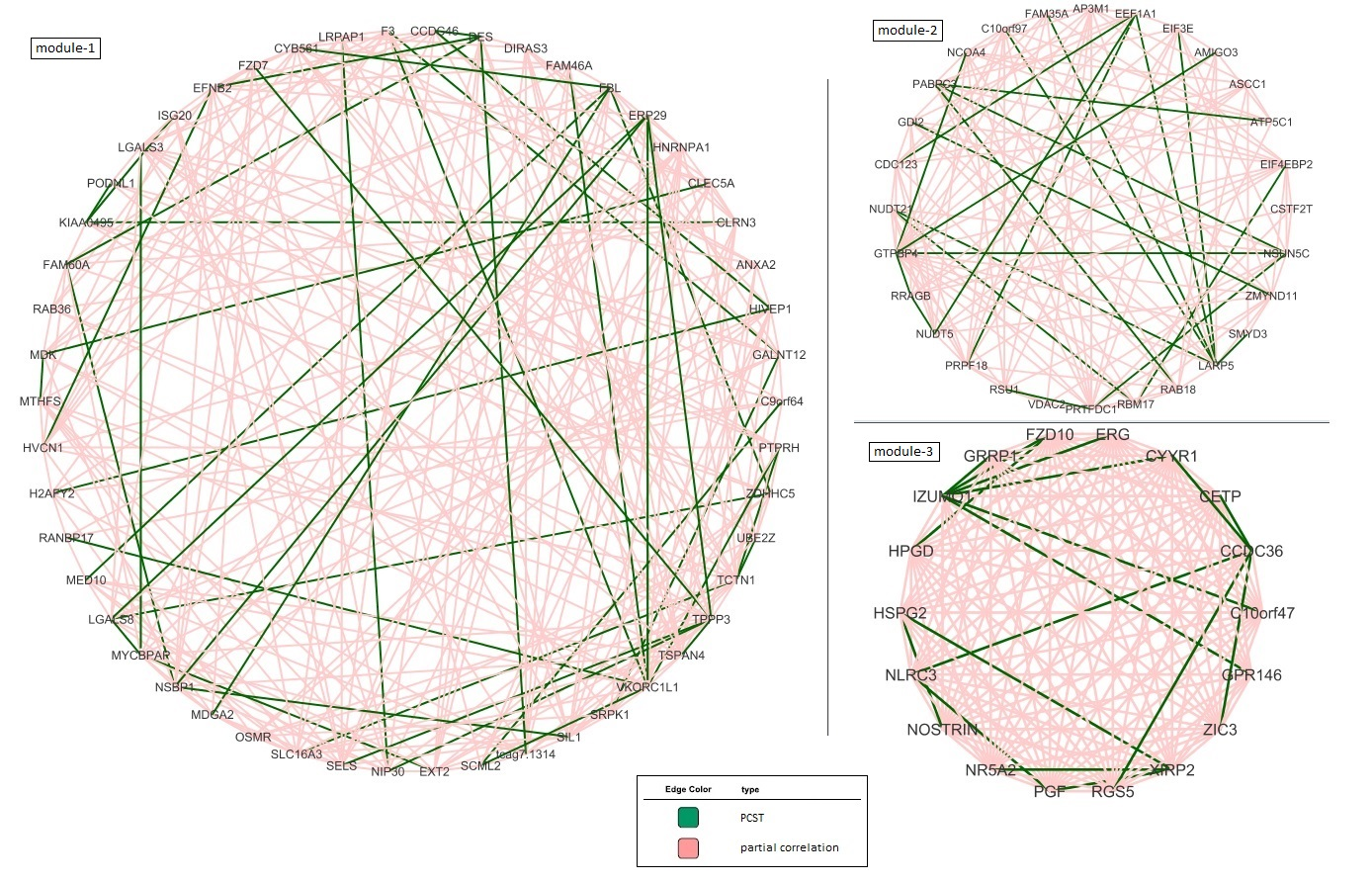


**Figure S1. The interaction modules inferred in partial correlation analysis and PCST.** Each pink edge represented the significant partial correlation (FDR<=0.05) between two genes. The green edges represented more reliable interactions that had the most evidence supporting their potential functionality in the cell.
