# Supplementary material for: A Systemic Analysis of Transcriptomic and Epigenomic Data To Reveal Regulation Patterns for Complex Disease
Source: G3 (Bethesda). 2017 May 11;7(7):2271–9. doi: 10.1534/g3.117.042408 (PMC5499134; doi:10.1534/g3.117.042408)
Supplement: Supplementary file 2 [file 2271FileS1.docx]

Supplementary File S1

**Robustness analysis**

We evaluate our framework in each of the layers, the WGCNA, FastGGM, PCST and SPLS respectively. We randomly divided our samples into 5 parts. Then we removed one part and used the remaining 4 parts of samples for the evaluation of each algorithm. This procedure was repeated 5 times to assess the reproducibility of the 3 modules with each part was removed once. We summarize the rate of the nodes that were identified at least 2 times for each module in the following table. Each layer showed high reproducibility (over 80% on average across 3 modules except the methylation sites in SPLS) as shown in Table S2.

**Table S2. Summary of the reproducibility for each layer**

|  |  | Module 1 | Module 2 | Module 3 | Average |
| --- | --- | --- | --- | --- | --- |
| WGCNA | Node | 87.69% | 77.78% | 94.12% | 86.53% |
|  | Total genes | 65 | 36 | 17 |  |
| FastGGM | Node | 100% | 100% | 100% | 100% |
|  | Total Nodes | 50 | 28 | 18 |  |
|  | Edge | 73.41% | 78.41 | 99.31% | 83.71% |
|  | Total Edges | 346 | 176 | 144 |  |
| PCST | Node | 100% | 100% | 100% | 100% |
|  | Total Nodes | 49 | 28 | 17 |  |
|  | Edge | 85.71% | 93.75% | 65.52% | 81.66% |
|  | Total Edges | 63 | 32 | 29 |  |
| SPLS | miRNAs | 85.11% | 88.23% | 67.50% | 80.28% |
|  | Total miRNAs | 47 | 17 | 40 |  |
|  | Methylation sites | 85.15% | 80.69% | 58.09% | 74.64% |
|  | Total Methylation sites | 377 | 290 | 272 |  |

The reproducibility of SPLS is lower than other layers. However, when inferring the regulation pattern of miRNAs and methylation sites on gene expression of each module, we incorporated the biological criteria for those miRNAs and methylation sites identified in SPLS. miRNA has been well known for the major function of cleaving transcripts of its target genes at the post-transcriptional level. Thus, the negative regulation between miRNA and gene expression is mainly focused in this study. Those miRNA-mRNA interactions with significant negative correlations and miRNA–target relationships are kept. In addition, DNA methylation is an important epigenomic mechanism to regulate gene expression. If there is a significant association for one gene between its methylation level and expression level, it is called *cis* relationship; otherwise it is called *trans* relationship. Since the reproducibility of *trans* relationships is still in debate, we will focus on genes with *cis* relationships. With these biological criteria, this layer also showed up to 100% reproducibility. Thus, these results indicated our framework can robustly yield reliable results.
